# Overexpression of *Ultrabithorax* Changes the Development of Silk Gland and the Expression of Fibroin Genes in *Bombyx mori*

**DOI:** 10.3390/ijms24076670

**Published:** 2023-04-03

**Authors:** Jiashuang Li, Yunhui Kong, Lingling Sun, Yaling Tang, Xia Sun, Sheng Qin, Muwang Li

**Affiliations:** 1 Jiangsu Key Laboratory of Sericultural Biology and Biotechnology, School of Biotechnology, Jiangsu University of Science and Technology, Zhenjiang 212018, China; 2 The Key Laboratory of Silkworm and Mulberry Genetic Improvement, Ministry of Agriculture, Sericultural Research Institute, Chinese Academy of Agricultural Science, Zhenjiang 212018, China

**Keywords:** Ubx, Myc, development, silk gland, *Bombyx mori*

## Abstract

*Ultrabithorax* (*Ubx*) is a member of the *Hox* gene group involved in cell fate decisions, cell proliferation and organ identity. Its function has been extensively researched in *Drosophila melanogaster* but little is known about it in Lepidoptera. To uncover the function of *Ubx* in the development of lepidopterans, we constructed the *Ubx* overexpression (Ubx^OE^) strain based on the Nistari strain of *Bombyx mori*. The Ubx^OE^ strain showed a small body size, transparent intersegmental membrane and abnormal posterior silk gland (PSG). In the current study, we focused on the effect of *Ubx* overexpression on the posterior silk gland. As the major protein product of PSG, the mRNA expression of *fibroin heavy chain* (*Fib-H*) and *fibroin light chain* (*Fib-L*) was upregulated three times in Ubx^OE^, but the protein expression of Fib-H and Fib-L was not significantly different. We speculated that the overexpression of *Ubx* downregulated the expression of *Myc* and further caused abnormal synthesis of the spliceosome and ribosome. Abnormalities of the spliceosome and ribosome affected the synthesis of protein in the PSG and changed its morphology.

## 1. Introduction

*Hox* genes, a subset of homeobox genes, play a core role in regulating insect body patterns along the head–tail axis. *Hox* genes were initially discovered in *Drosophila melanogaster* (*D. melanogaster*), but it was later discovered that they can also control body morphology in mammals, including *Mus musculus* and *Homo sapiens* [1]. *Hox* genes are a group of transcription factor genes encoding a DNA binding motif containing 60 amino acids (Homeodomain), which is remarkably conserved throughout evolution [2,3,4]. *Ubx*, a member of the *Hox* gene group that interacts with *Exd* and *Hth*, has different functions in different regions of the wing imaginal disc in *D. melanogaster* [5,6]. The *Hox* genes of *Bombyx mori* (*B. mori*) and the *Hox* genes of *D. melanogaster* show high structural and sequential homology.

*Ultrabithorax* (*Ubx*) is a member of the bithorax complex [7,8,9] (consisting of *Ultrabithorax*, *Abdominal A* and *Abdominal B* in *B. mori*) [10], which plays a prominent role in the body plan. In *D. melanogaster*, *Ubx* plays a significant role in many areas. For instance, *Ubx* regulates forewing/hindwing differentiation [11] and is involved in autophagy [12] and the splicing biological pathway [13]. However, there have been few studies on *Ubx* in *B. mori*, although it is known that knockdown of *Ubx* impacts the development of the embryo [14].

*Myc* is targeted by Ubx, according to previous ChIP-seq [15], which was performed in wing discs of *B. mori* and *D. melanogaster*. *Myc* is a central regulator of growth and/or proliferation of many cell types [16], such as imaginal disc cells [17], polyploid cells [18,19], stem cells [20,21] and blood cells [22]. As an oncogene in mammalian tumour cells [23], *Myc* takes part in cell proliferation and differentiation, mainly by affecting DNA replication and the transition from the G1 to S phase [24,25,26]. Previous studies in *B. mori* also confirmed that *Myc* is involved in the regulation of the cell cycle and DNA replication [27].

In *B. mori*, knockdown of *Ubx* at the embryonic stage causes an additional pair of thoracic leg-like protuberances in A1 [14]. However, the actual functions of *Ubx* remain unclear. In this study, the overexpression of *Ubx* in *B. mori* affected the shape of the posterior silk gland (PSG), the transparency of the intersegmental membrane and the body size of the pupa. The production of fibroin heavy chain and light chain increased, but there was no significant effect on the production of silk protein. It is possible that the overexpression of *Ubx* inhibited *Myc* and related biological processes including ribosome biogenesis and the spliceosome. Our results suggest that *Ubx* plays a crucial role during the development of *B. mori*, especially of the silk gland. 

## 2. Results

### 2.1. Construction of Ubx Overexpression Strain

**Figure 1:**
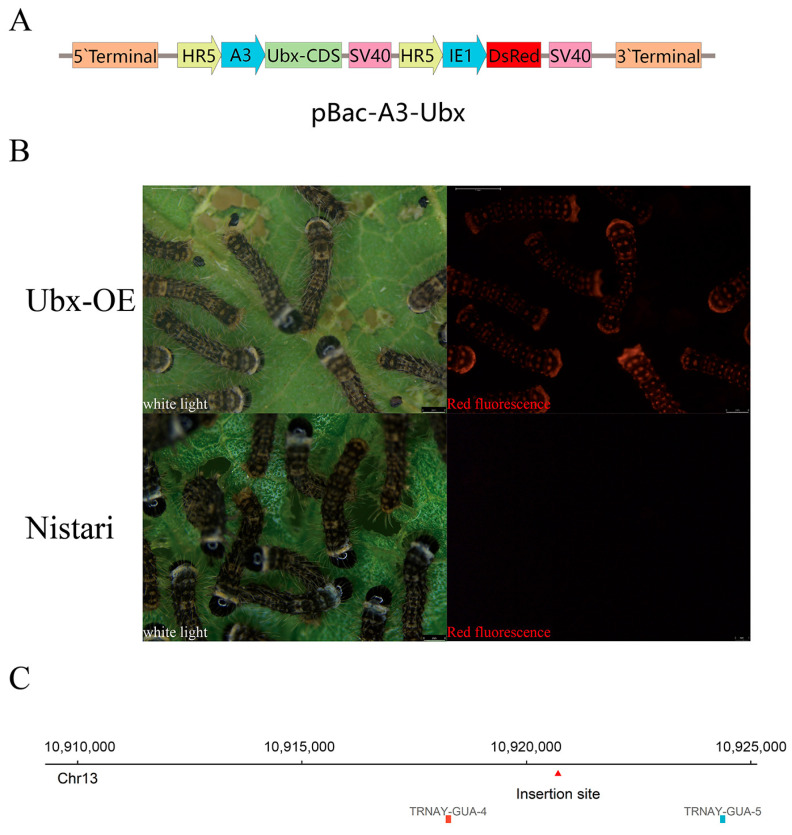
Construction of overexpressed *Ubx* silkworm. (**A**) The schematic diagram of *Ubx* overexpression plasmid. A3-promoter, the promoter of *Actin3*; Ubx-CDS, the coding sequence of *Ubx*; DsRed2, the red fluorescent protein gene. (**B**) The ant silkworms of Ubx^OE^ with red fluorescent protein (RFP) gene and Nistari (no RFP) under white light conditions and 532 nm red fluorescent excitation light. The scale bar in the lower right of pictures shows 1 mm. (**C**) Relative position of *Ubx* overexpression plasmids inserted site (red arrow) on genome of *Bombyx mori*. Ubx-OE, the transgenic strain with *Ultrabithorax* overexpressed, namely Ubx^OE^. Nistari, the control of Ubx^OE^.

The whole coding sequence (CDS) of *Ubx* was cloned from the PSG of Nistari and was the same as the sequence in the NCBI (ID: NM_001114160.1). The structure of the plasmid for overexpressing *Ubx* is shown in Figure 1A. The plasmid was injected into eggs of Nistari. Transgenic individuals were screened using the red fluorescent labelling protein (Figure 1B).

To detect whether the imported sequence interrupted other genes, we detected the insertion site of the *Ubx* overexpression plasmid in the silkworm genome using inverse PCR. The PCR results showed one insertion site at 10,920,272 bp of chromosome Chr13 (Figure 1C, details in Figure S1). Within 5 kb upstream and downstream of the insertion site, there were only two tRNAs but no coding gene. This suggested that the insertion of the *Ubx* overexpression plasmid had no influence on known coding genes in our Ubx^OE^ strain.

**Figure 2:**
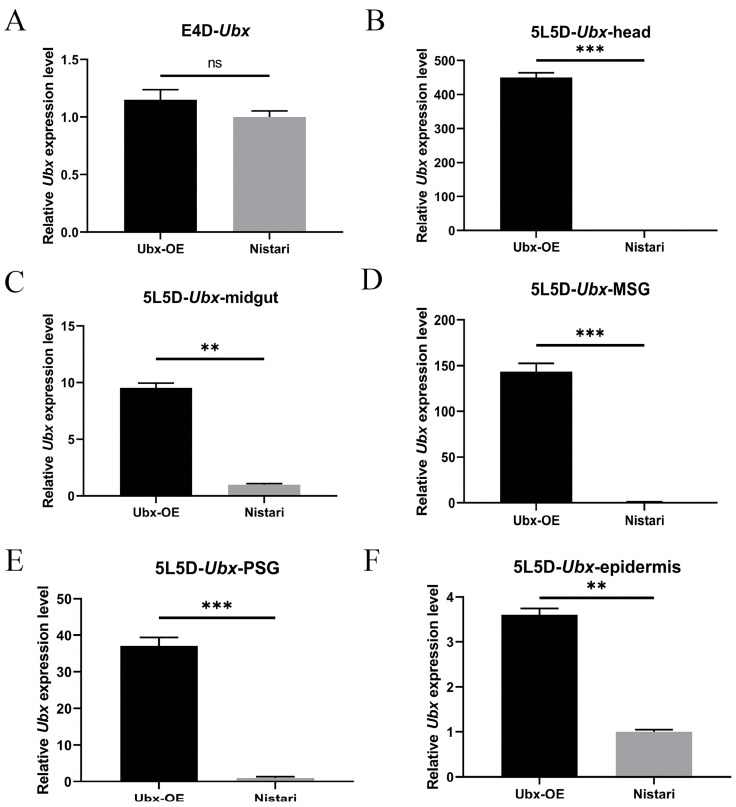
The relative expression level of Ubx in different tissues of UbxOE and Nistari silkworms. (**A**) The expression level of Ubx in eggs of Ubx^OE^ and Nistari on the fourth day of embryonic stage (E4D). (**B**) The difference in Ubx expression level in head on the fifth day of fifth instar (5L5D). (**C**) The difference in Ubx expression level in midgut on 5L5D. (**D**) The difference in Ubx expression level in middle silk gland on 5L5D. (**E**) The difference in Ubx expression level in posterior silk gland on 5L5D. (**F**) The difference in Ubx expression level in epidermis on 5L5D. Ubx-OE, the transgenic strain with *Ultrabithorax* overexpressed, namely Ubx^OE^. Nistari, the control of Ubx^OE^. Error bars indicate the standard error of the mean (*n* = 3). Significant differences were assessed using Student’s *t*-test (ns *p* > 0.05, ** *p* < 0.01, and *** *p* < 0.001).

To confirm whether *Ubx* was successfully overexpressed in transgenic individuals, we compared the expression levels in Ubx^OE^ and Nistari strains in the embryo and fifth-instar stages. Given the important role of Ubx in the development of the embryo, we collected 4-day-old eggs from Ubx^OE^ and Nistari. Each group contained three samples and each sample contained 30 eggs. The qRT-PCR results showed that although the expression of *Ubx* in Ubx^OE^ was slightly higher than that in Nistari, the difference was no more than 1.5 times. There was no statistically significant difference between Ubx^OE^ and Nistari (Figure 2A). It has been demonstrated that the A3 promoter can activate the expression of exogenous genes at the embryo stage [28,29]. Therefore, we inferred that the inactivated overexpression of *Ubx* might be related to the epigenetic modification and chromatin activity [11]. Next, we chose to detect the expression levels of *Ubx* at the larval stage. It was extremely highly expressed in the head, midgut, middle silk gland, posterior silk gland and epidermis of Ubx^OE^ fifth-instar larvae (Figure 2B–F). According to the expression patterns of *Hox* genes, *Ubx* should not be expressed in the anterior of the body (e.g., head); however, expression was enhanced in the head of Ubx^OE^ larvae. In the middle silk gland of Ubx^OE^, its expression level was 140 times higher than that of the wild type. In the posterior silk gland where *Ubx* was originally expressed, the overexpression of *Ubx* was nearly 40-fold. *Ubx* expression also increased in some other tissues at the fifth-instar stage, but with a low and non-significant fold change.

### 2.2. Phenotype of UbxOE Strain

**Figure 3:**
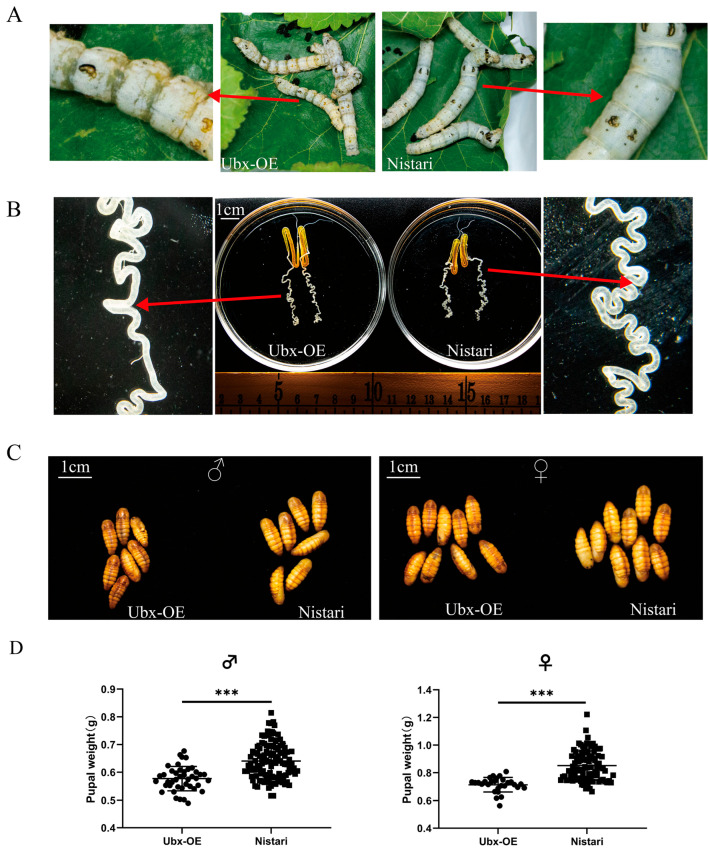
The phenotype changes between Ubx^OE^ and Nistari. (**A**) Intersegmental membrane of fifth-instar larva of Ubx^OE^ and Nistari. (**B**) The posterior silk gland on fourth day of fifth instar (5L5D) of Ubx^OE^ and Nistari. (**C**) The male and female pupae of Ubx^OE^ and Nistari. (**D**) The male and female pupal weight of Ubx^OE^ and Nistari. The scale in bar the upper left of pictures shows 1 mm. Ubx-OE, the transgenic strain with *Ultrabithorax* overexpressed, namely Ubx^OE^. Nistari, the control of Ubx^OE^. Statistical samples > 30. Significant differences were assessed using Student’s *t*-test (*** *p* < 0.001).

Ubx^OE^ and Nistari showed different phenotypes at the larval stage (Figure 3). Firstly, the Ubx^OE^ larvae were thinner than the Nistari larvae and the intersegmental membrane was transparent (Figure 3A). The posterior silk gland of Ubx^OE^ was shorter and had less curvature than that of Nistari (Figure 3B). In addition, the pupa of Ubx^OE^ was smaller and thinner than that of Nistari, and this difference was more significant in the female pupae (Figure 3C). The pupal weight of Ubx^OE^ was significantly lighter than that of Nistari, in both males and females (Figure 3D). These changes in phenotype suggest that the overexpression of *Ubx* influences the development of silkworms.

### 2.3. Overexpression of Ubx Upregulated the Expression of Fibroin Genes but Inhibited the Synthesis of Fibroin Protein

**Figure 4:**
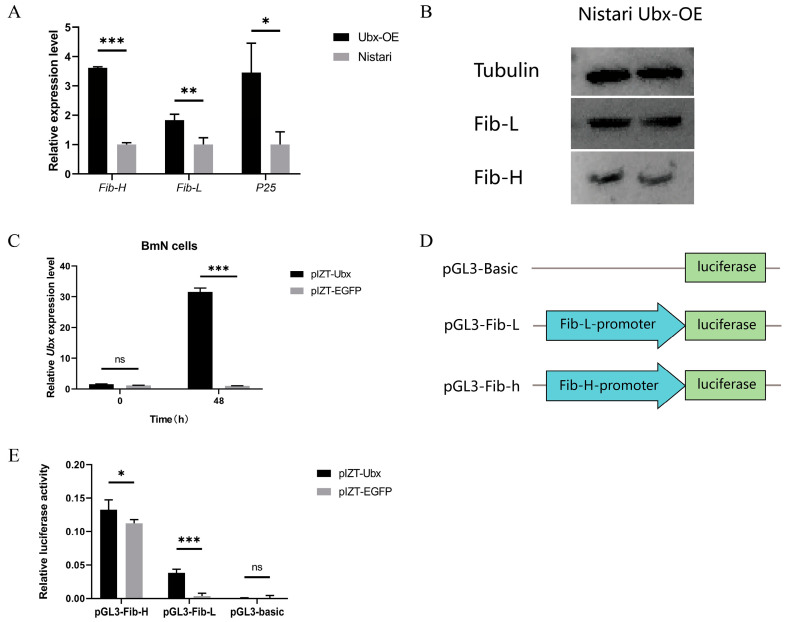
Activation effect of promoter of fibroin genes by Ubx. (**A**) The expression of fibroin genes in Ubx^OE^ and Nistari. *Fib-H* means *fibroin heavy chain* and *Fib-L* means *fibroin light chain*. (**B**) Western-blot result of Fib-L and Fib-H in the PSG of third-day fifth-instar larva of Ubx^OE^ and Nistari. (**C**) Overexpression of *Ubx* in BmN cells. The expression of *Ubx* was significantly enhanced by pIZT-Ubx at 48 h after transfection. The pIZT-EGFP was used as a negative control. (**D**) Design of reporter plasmids in dual luciferase reporter assay. (**E**) Promoter activity of *Fib-L* and *Fib-H* after overexpressing *Ubx* in BmN cells. Ubx-OE, the transgenic strain with *Ultrabithorax* overexpressed, namely Ubx^OE^. Nistari, the control of Ubx^OE^. pIZT-Ubx, BmN cells transfected with plasmid of pIZT-mCherry-V5-his-Ubx, overexpressed *Ultrabithorax*. pIZT-EGFP, BmN cells transfected with plasmid of pIZT-mCherry-V5-his-EGFP, was the negative control of pIZT-Ubx. Error bars indicate the standard error of the mean (*n* = 3). Significant differences were assessed using Student’s *t*-test (ns *p* > 0.05, * *p* < 0.05, ** *p* < 0.01, and *** *p* < 0.001).

We observed an abnormality in the posterior silk gland of Ubx^OE^. This might be associated with the content of fibroin proteins. Fibroin protein is composed of fibroin heavy chain (Fib-H), fibroin light chain (Fib-L) and P25 protein. We detected the mRNA expression level of *Fib-H*, *Fib-L* and *P25* in the PSG of three-day-old fifth-instar Ubx^OE^ larvae, and found that Ubx upregulated the mRNA expression level of these fibroin genes (Figure 4A). RNA-seq data also supported this result (Section 2.4 and Table S1). However, Western blot analysis showed that there was no significant difference in protein level (Figure 4B). This indicates that the overexpression of *Ubx* upregulated the expression of fibroin genes but not the synthesis of fibroin protein in Ubx^OE^. To further verify whether the overexpression of *Ubx* can activate the expression of *Fib-H* and *Fib-L*, *Ubx* was overexpressed in BmN cells and a dual-luciferase reporter (DLR) assay system was constructed, including the promoter of *Fib-H* and *Fib-L* (Figure 4D). BmN cells derived from the ovaries of *B. mori* do not express fibroin genes. After overexpressing *Ubx* (Figure 4C), the expression of *Luciferase* was initiated by the *Fib-L* promoter and the fluorescence signal was significantly enhanced, but that of the *Fib-H* promoter only increased slightly (Figure 4D,E). This implies that *Fib-L* might be activated by Ubx.

### 2.4. Transcriptome of PSG Revealed Differences in mRNA Level

**Figure 5:**
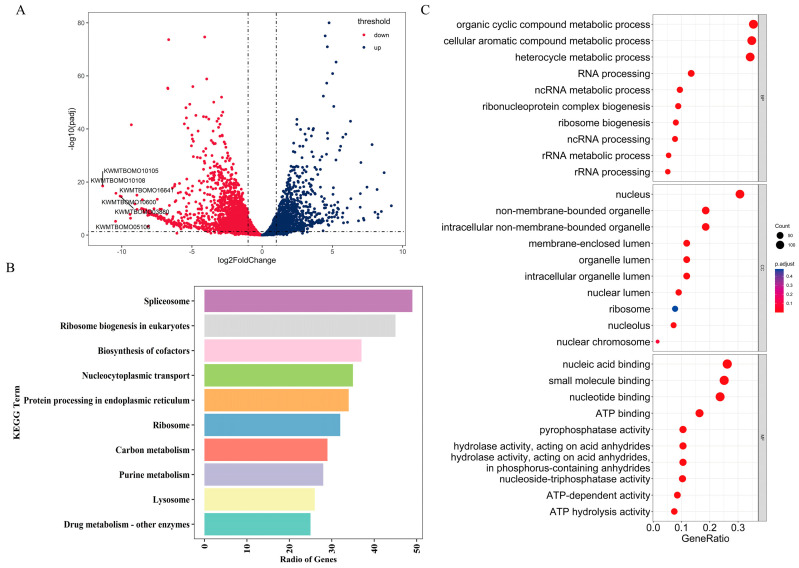
Transcriptome analysis of DEGs between Ubx^OE^ and its Nistari. (**A**) Volcano map of DEGs between Ubx^OE^ and Nistari. (**B**) KEGG enrichment analysis of DEGs between Ubx^OE^ and Nistari. (**C**) GO enrichment analysis of DEGs between Ubx^OE^ and Nistari.

To explore how *Ubx* overexpression affects the development of the posterior silk gland, we compared the differences in transcriptome between Ubx^OE^ and Nistari. In total, 2904 differentially expressed genes were identified. Compared to Nistari, 1213 genes were upregulated and 1691 genes were downregulated in the posterior silk gland of three-day-old fifth-instar larvae (Figure 5A, Table S1). After functional annotation and pathway enrichment analyses, 10 KEGG pathways and 56 enriched GO terms were screened (Figure 5B,C, Table S2).

Most notably, we found that the results of GO enrichment analysis and KEGG enrichment analysis showed that most of the differentially expressed genes (DEGs) in *Ubx* overexpressed tissues were related to protein synthesis (Figure 5B,C), suggesting that the abnormality of silk glands may be related to protein synthesis.

### 2.5. Identification of DEGs Potentially Regulated by Ubx and Myc

**Figure 6:**
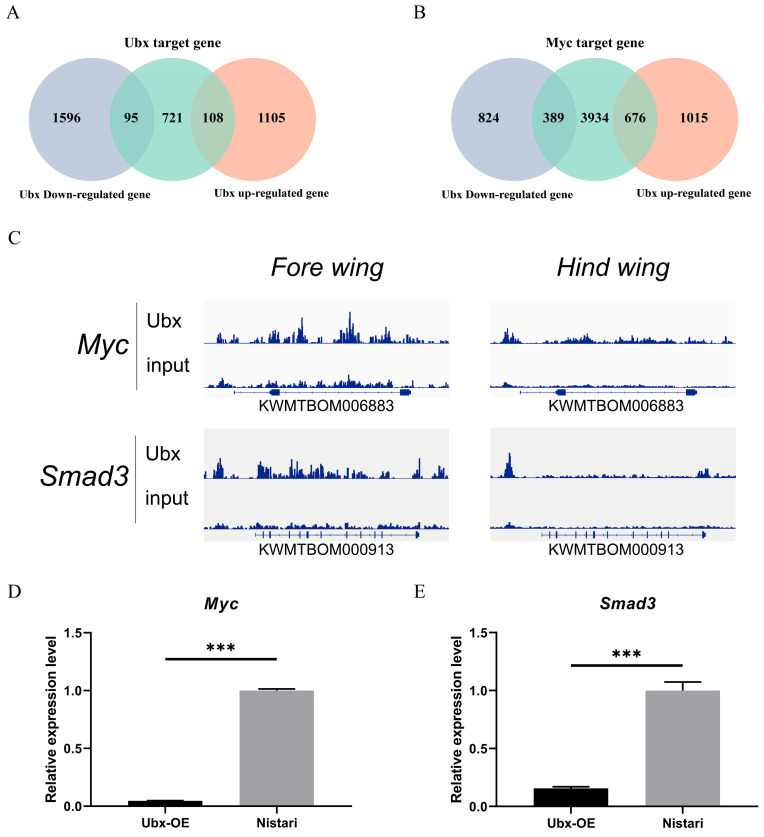
Target DEGs of Ubx and Myc. (**A**) Venn diagram of target genes of Ubx and DEGs. (**B**) Venn diagram of target genes of Myc and DEGs. (**C**) The binding peak map of Ubx in the *Myc* (ID: KWMTBOM006883) and *Smad3* (ID: KWMTBOM000913) gene regions from ChIP-seq in the fore wing and hind wing of *B. mori*. (**D**) Expression of *Myc* in Ubx^OE^ and Nistari. (**E**) Expression of *Smad3* in Ubx^OE^ and Nistari. Ubx-OE, the transgenic strain with *Ultrabithorax* overexpressed, namely Ubx^OE^. Nistari, the wild type of Ubx^OE^. Error bars indicate the standard error of the mean (*n* = 3). Significant differences were assessed using Student’s *t*-test (*** *p* < 0.001).

As a transcription factor, Ubx plays a crucial role in the regulation of transcription [30,31]. To identify the genes potentially regulated by Ubx directly, we performed an analysis combining RNA-seq data and ChIP-seq data (Figure 6). Using the ChIP-seq data, 924 genes were identified as Ubx targets (Figure 6A and Figure S2). Compared to RNA-seq, 203 DEGs out of 924 Ubx targets were identified (Table S3). In total, 108 genes were upregulated and 95 genes were downregulated in the PSG of the Ubx^OE^ strain.

We analysed the function of DEGs targeted by Ubx based on GO and KEGG (Figure S3, details in Table S4). The GO enrichment results showed that the function of DEGs targeted by Ubx is mainly related to “purine nucleotide binding”, “DNA-binding transcription factor activity”, “nucleoside-triphosphatase activity”, “pyrophosphatase activity” and “hydrolase activity”. These DEGs participate in RNA, ncRNA, rRNA processing, and ribosome biogenesis. The KEGG enrichment analysis suggested that DEGs targeted by Ubx are mainly involved in “protein processing in endoplasmic reticulum”, “apoptosis”, “phagosome”, the “MAPK signalling pathway”, the “FoxO signalling pathway”, “mitophagy” and the “Hippo signalling pathway”.

However, DEGs potentially regulated by Ubx only accounted for 7.92% (230/2904) of all DEGs. Their annotation was also different from the annotation of all DEGs. *Myc*, a crucial transcription factor, can potentially regulate 1065 DEGs, according to ChIP-seq data for the *D. melanogaster* homologue, accounting for 36.67% (1065/2904) of all DEGs (Table S5). The RNA-seq and qRT-PCR results showed that it was significantly downregulated in the Ubx^OE^ strain (Figure 6D). The expression level of *Myc* in Nistari was approximately eight times higher than that of Ubx^OE^. To further confirm the regulation effect of *Ubx* on *Myc*, we knocked down the expression of *Ubx* in BmN cells and detected the change in *Myc* expression using qPCR. The result showed that the downregulation of *Ubx* enhanced the expression of *Myc* (Figure S4). This is consistent with the observation at the individual level. Pathway analysis showed that it can be potentially regulated by the TGF-beta signalling pathway (Figure S5), Wnt signalling pathway (Figure S6), Hippo signalling pathway and MAPK pathway. Meanwhile, we also observed that the expression of *Smad3*, an upstream gene of *Myc* in the TGF-beta signalling pathway and Wnt signalling pathway, was also strongly inhibited in Ubx^OE^ to approximately 10% of that in Nistari (Figure 6E).

We further analysed the function of DEGs potentially targeted by *Myc*. The GO and KEGG enrichment results showed high consistency for all DEGs (Figure S7, details in Table S6). In the top ten GO enrichment terms of DEGs targeted by Myc, all biological process terms, 90% of the cellular component terms and half of the molecular function terms were found in all DEGs. The top four KEGG pathways enriched in Myc target DEGs were also found in all DEGs. This result suggests that a large proportion of changes caused by overexpressing Ubx at the transcriptome level might act via the inhibition of *Myc*.

## 3. Discussion

*Hox* genes regulate the expression of many genes that produce transcription factors and determine the specificity of body segments in insects and mammals, and even play an important role in humans [32,33,34]. *Ultrabithorax* is a member of the bithorax complex and features in body segment specificity [11,35]. In this study, we constructed a *Ubx* overexpression silkworm using the piggyBac transposon system and named the resulting strain Ubx^OE^. Ubx^OE^ showed a series of phenotypes different from the wild type such as small larvae, small pupae and a shorter PSG with less curvature.

**Figure 7:**
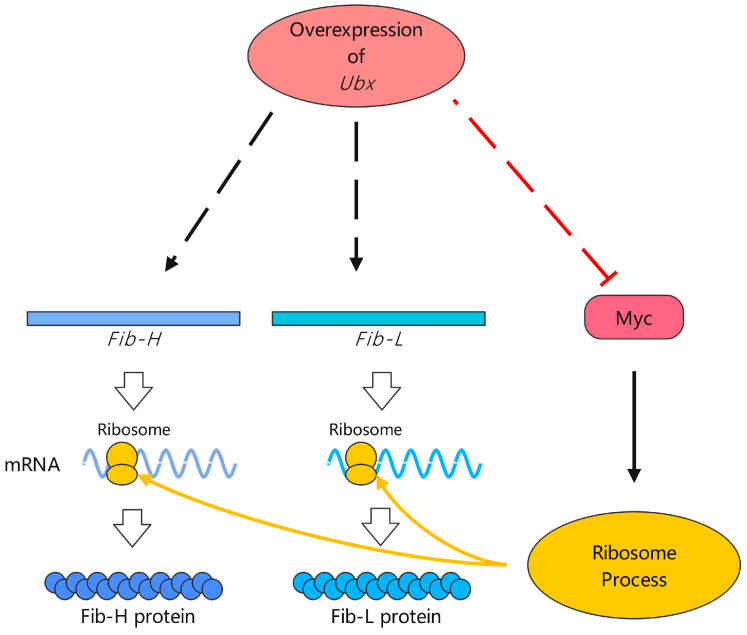
Presumptive model of the expression of fibroin regulated by overexpressing *Ubx*. Overexpression of *Ubx* increased expression of *Fib-H* and *Fib-L* but inhibited the expression of *Myc*. Decrease in *Myc* impacted the synthesis of ribosomes. Therefore, the mRNA of *Fib-H* and *Fib-L* was increased but the content of fibroin protein was not significantly changed. Arrow indicates activation and “T” indicates inhibition. Solid line indicates direct regulation. Dashed line indicates indirect regulation or indeterminate direct regulation.

A previous study demonstrated that *Hox* genes participate in the development of the silk gland during embryonic and larval development. The expression of *Antp* was restricted in the middle silk gland (MSG), whereas *Ubx* was specifically expressed in the PSG [36]. This indicated that *Ubx* might be involved in regulating the expression of genes in the PSG. Our results demonstrated that overexpressing *Ubx* increased the expression of *Fib-H* and *Fib-L*. Although we speculated that the expression of *Fib-H* and *Fib-L* was enhanced after overexpressing *Ubx*, the content of fibroin protein in Ubx^OE^ did not differ significantly from the wild type. This may be due to the low expression of *Myc* (Figure 7). We observed that overexpressing *Ubx* significantly inhibited the expression of *Myc* in the PSG. According to the GO and KEGG pathway analyses, the downregulation of *Myc* would impact the synthesis of the spliceosome and ribosomes that participate directly in the synthesis of fibroin proteins. Meanwhile, aberrantly expressed spliceosome and ribosomes also affect the synthesis of other proteins in the cells of PSG. The reduction in protein content would lead to a shorter PSG. Other evidence also suggests that the overexpression of *dMyc* can increase nucleolar size and cellular ribosome content in the salivary gland of *D. melanogaster* [37]. The overexpression of *Myc* enhanced the DNA replication and synthesis of silk protein in the silk gland of *B. mori* [27]. This proves our viewpoint from the opposite side. A well-known function of *Myc* is that it is involved in controlling organism size. For example, overexpressing *dMyc* increased the adult size of *D. melanogaster* while the hypomorphic *dMyd* mutation resulted in small adult flies [17,38]. In our research, the small body size of Ubx^OE^ might also be related to the low expression of *Myc*.

Although we demonstrated that the expression of *Myc* was significantly inhibited in Ubx^OE^, the details are still unknown. Many regulators can activate or inhibit the expression of *Myc*. A famous pathway is the TGF-beta signalling pathway in which Smad3 activates p107, E2F4/5 and DP1 to inhibit the expression of *Myc* [39]. However, our result showed that both *Smad3* and *Myc* were downregulated in the Ubx^OE^ strain. The expression of *Myc* can also be activated by Yki/Sd via the Hippo signalling pathway [40]. However, we found that the expression of *Yki* and *Sd* was not significantly different between Ubx^OE^ and Nistari. Previous studies showed that LEF/TCF can activate *Myc* via the Wnt signalling pathway in mammals but there is still no evidence for it in insects [41,42].

Another possible explanation for the formation of a shorter PSG is a reduction in the number of PSG cells. The cell number in the silk gland is determined at the early and middle embryonic stages in *B. mori* [43]. A previous study reported that knockdown of *Bmsage* and *BmDfd* at the early embryonic stage decreased the number of cells in the PSG [44,45]. However, the effect of overexpressing *Ubx* on the morphology of the silk glands may not have been apparent at the embryonic stage in our study. In *B. mori*, the silk gland was formatted in the embryonic stage on the fourth day [46]. We detected the expression level of *Ubx* to explore whether overexpressing *Ubx* affects the development of the embryo. Contrary to expectations, the overexpression of *Ubx* had no significant effect in Ubx^OE^ embryos. This may be associated with H3K27me3 modification at the embryonic stage [47]. Our inserted *Ubx* was not activated at the embryonic stage. Based on the above analysis, we suggest that abnormalities in the PSG were more likely due to the downregulation of *Myc* caused by the overexpression of *Ubx*.

## 4. Materials and Methods

### 4.1. Silkworm Strains

The non-diapause silkworms of the strain Nistari were obtained from the Sericulture Research Institute at the Chinese Academy of Agriculture Science, Zhenjiang, Jiangsu province. The silkworm eggs were cultured at a standard temperature of 25 °C under a photoperiod of 12 h light and 12 h dark. The larvae were reared on fresh mulberry leaves and maintained in a 12 h light and 12 h dark cycle at 25 °C with 80 ± 5% relative humidity.

### 4.2. Plasmids in This Study and Constructions of Transgenic Strain

The whole coding sequence of *Ubx* was cloned from RNA that was extracted from the posterior silk gland of Nistari. The piggyBac-based transgenic plasmid of pBac-DsRed-A3-Ubx was used to construct the overexpression strain of *Ubx*. The pBac-DsRed-A3-Ubx plasmid was microinjected into 0th generation (G_0_) eggs of Nistari, which were then incubated at 25 °C in a humidified chamber until hatching. The G_1_ silkworms were obtained from G_0_ selfing. After hatching of the G_1_ generation, successful transgenic silkworms were screened based on the DsRed protein carried on the plasmid. The plasmids of pIZT-mCherry-Ubx, pIZT-mCherry-V5-his-EGFP, pGL3-Fib-L, PGL3-Fib-H and pGL3-basic were used to verify the promoter activity in the BmN cells that were overexpressing *Ubx*.

### 4.3. Detection of Plasmid Insertion Site

Genome DNA was extracted from the PSG of the Ubx^OE^ strain in third-day fifth-instar larva, and its quality is tested with a Nanodrop1000 (Thermo Fisher Scientific, Waltham, MA, USA). The genome was extensively digested by DpnⅡ (NEB, Ipswich, MA, USA) and purified immediately using an AxyPrep PCR cleanup Kit (Axygen, Corning, NY, USA). The recovered fragments were cyclized using T4 ligase (Takara, Kusatsu, Japan). The cyclized product was used as a template, which used a pair of primers set inside the plasmid, for amplification. The primers are listed in Table S7. Finally, the amplified fragments were sequenced and aligned to the genome using NCBI BLAST to obtain the genome location.

### 4.4. Cell Culture, Transfection and Dual Luciferase Reporter Assay System

The BmN cells (provided by the Sericulture Research Institute at the Chinese Academy of Agriculture Science) were cultured in TC-100 insect medium (AppliChem, Darmstadt, Germany) that contained 10% foetal bovine serum (Gibco, Life technologies, New York, NY, USA) and 1% penicillin–streptomycin solution (Gibco) at 27 °C. 

The plasmid of pIZT-mCherry-V5-his-Ubx was transfected into BmN cells using the Neofect DNA Transfection Reagent (Neofect biotech, Beijing, China) as described in the protocol, and the plasmid of pIZT-mCherry-V5-his-EGFP was used as a negative control. Cells were collected separately 0 h and 48 h after transfection. Collected cells were washed with PBS and soaked in Trizol (Takara, Japan) for storage at −80 °C.

For the dual luciferase reporter assay (DLR) experiment, pIZT-mCherry-V5-his-Ubx and pIZT-mCherry-V5-his-EGFP were separately co-transfected in BmN cells with pGL3-Fib-L, pGL3-Fib-H and pGL3-basic (negative control without promoter regions) plasmids (2 × 3 = 6 groups in total, with 3 replicates for each group) and the PRL-CMV plasmid as an internal reference in each repeat. The intensity of luciferase was detected with the Dual-Luciferase Reporter Assay System (Promega, Madison, WI, USA) and GLOMAX 20/20 LUMINOMETER (Promega) as described in the protocol.

### 4.5. Transgenic Strain Phenotypic Analysis

Through the microinjection technique, we obtained a *Ubx* overexpression transgenic strain and named it Ubx^OE^. For silk glands, silkworms on the 4th day of the fifth larva were dissected to obtain their complete silk glands. The silk glands were placed in phosphate-buffered saline and photographed after dissecting. Statistical differences were evaluated using Student’s *t*-tests for unpaired samples. The level of statistical significance was set as follows: * *p* < 0.05, ** *p* < 0.01, and *** *p* < 0.001.

### 4.6. RNA Extraction for qRT-PCR

Total RNA of Nistari and Ubx^OE^ was extracted as described in the manual of Trizol (Takara). The RNA was then reverse-transcribed into cDNA using a PrimeScript RT reagent Kit with gDNA Eraser (Perfect Real Time) (Takara). The cDNA can only be used after the amplification of internal reference genes is verified to be free of problems. The qRT-PCR experiment was performed using a NovoStart SYBR qPCR SuperMix (Novoprotein, Beijing, China). The data were processed using lightcycler96 software (Roche, Basel, Switzerland) based on the 2^−ΔΔct^ method. All primers for qRT-PCR are listed in Table S7. The gene expression levels were compared using Student’s *t*-tests.

### 4.7. RNA Library Construction for RNA-Seq

Posterior silk glands in fourth-day fifth-instar larva were applicated in the RNA-seq. After RNA extraction, a Fragment Analyzer 5400 (Agilent Technologies, Santa Clara, CA, USA) was used to ensure the integrity of total RNA. A NEBNext UltraTM RNA Library Prep Kit for Illumina (NEB) was used to generate the sequencing libraries as per the manufacturer’s recommendations, PCR products were purified (AMPure XP system) and library quality was assessed using the Agilent Bioanalyzer 2100 system. The cDNA libraries were sequenced on an Illumina Novaseq 6000 platform and paired-end reads with a length of 150 bp were generated. The sequencing was performed using Novogene (Beijing, China).

### 4.8. Analyzing Ubx Target Genes Based on ChIP-Seq and RNA-Seq Data

Cleaned reads were mapped to a reference genome from Kaikobase using hisat2 [48,49], and FeatureCounts was used to perform gene expression quantification [50]. DEseq2 was used to perform differential gene expression analysis [51]. We defined genes with |log2 Fold Change (Log_2_ FC)| ≥ 1 and adjusted *p* value ≤ 0.05 as differentially expressed genes (DEGs), which were used to perform GO and KEGG enrichment analysis with the R package ClusterProfiler [52].

For ChIP-seq, raw data of *B. mori* Ubx were obtained from the NCBI SRA database under the accession PRJNA292691. The peak calling was performed using MACS2, while HOMER was used for motif analysis [53]. The GO and KEGG enrichment analysis were performed in the same way as RNA-seq. Finally, the peak of the hind wing and fore wing were merged for conjoint analysis with RNA-seq to compare the similarities and differences between RNA-seq and ChIP-seq [54].

To identify the target DEGs of Myc, the target gene of Myc of *D. melanogaster* was obtained from the ChIP-Atlas and converted with the corresponding silkworm homolog by Orthodb [55,56]. The circus plot was visualized with the R package circlize [57].

### 4.9. Protein Extraction and Western-Blot

Total proteins were extracted from the 3rd day of 5th larva using lysis buffer with prohibitor and PMSF. The mixture of ground PSG and lysis buffer was purified by centrifugation after stewing for 30 min. A Bradford Protein Assay Kit (Sangon, Shanghai, China) was used to detected the concentration of protein solution right after the centrifugation. The supernatant was added with 5× loading buffer with a volume of 1/5 then the sample was boiled for 10 min and stored at −80 °C. Samples were separated using SDS-PAGE at 80 V for 0.5 h and 120 V for 1 h, and then transferred to PVDF membranes. The primary antibodies of Fib-L and Fib-H (obtained from Professor Tan Anjiang, Science of Plant Physiology and Ecology, Chinese Academy of Sciences [58]) were incubated with target proteins, and tubulin antibody (1:4000, Bioss ANTIBODIES, Beijing, China) was used as an internal reference. Additionally, secondary antibody (HRP Goat Anti-Rabbit IgG, 1:5000, ABclonal Technology, Wuhan, China) was incubated with PVDF membranes combined with the first antibody. The membranes were incubated with an eECL Western Blot Kit (Vazyme, Nanjing, China) and photographed in the ChemiScope Western Blot Imaging System (CLINX, Shanghai, China).

## Data Availability

The transcriptome data in this study have been uploaded to NCBI with the serial number PRJNA905723. The analysed documents have been uploaded as Supplementary Materials.

## Supplementary Materials

The following supporting information can be downloaded at: https://www.mdpi.com/article/10.3390/ijms24076670/s1.
