# Supplementary material for: Overexpression of Ultrabithorax Changes the Development of Silk Gland and the Expression of Fibroin Genes in Bombyx mori
Source: Int J Mol Sci. 2023 Apr 3;24(7):6670. doi: 10.3390/ijms24076670 (PMC10095271; doi:10.3390/ijms24076670)
Supplement: Supplementary file 1 [file ijms-24-06670-s001.zip › jpm-2298695-tex.zip!/Definitions/logo-ccby-eps-converted-to.pdf]

CC BY
